# The Institutional Library

**Published:** 1917-06-02

**Authors:** 


### THE INSTITUTIONAL LIBRARY.

### SCIENCE AND THE NATION.

**Science and the Nation.** Essays by Cambridge Graduates, with an Introduction by the Right Hon. LORD MOULTON. (Cambridge University Press. 1917. Price 5s. net.)

LORD MOULTON clearly indicates the purpose of the interesting set of essays procured by Dr. Seward from some of his colleagues at Cambridge. It is a defect of party Government, much in evidence in this country, that our political rulers clutch the pulse of the public as eagerly as a man about to drown clutches a straw. The Great Panjandrums have to consider the political machine, for in that and by that they have their being and their greatness. Recent events have set the public pulse throbbing to some purpose. It has been realised that England was dependent on Germany for a large number of processes and products of vital importance, and that she had not sufficient trained experts to "carry on," quite apart from improving the processes and adding to the products. In a word, England had been neglecting science. The politicians suddenly began to sit up and take notice, notice of what scientific men have been preaching to deaf ears for many a generation. They had received what they call a "mandate," and so something had to be done for science.

What is going to be done is that more money, contributed partly by manufacturers and partly by the State, ever fond of the "grant in aid," is to be expended on applied science. The politicians appear to argue that as what we need is more dyes and drugs, better technical methods in manufacture, improvements in agriculture, the direct way to obtain these results is to set people down to "apply" science, to make more dyes and drugs, improve technical methods in manufactures and agriculture. The object of the authors of these essays is to show that this, if not altogether wrong, is a very imperfect method.

All the writers are distinguished experts in their subjects—in chemistry, physics, metallurgy, mathematics, botany, forestry, plant breeding, agriculture, geology, various branches of experimental medicine, anthropology. They all insist from the point of view of theory, and prove by abundant examples, that most of the great discoveries which have changed the modern world, have added countless millions to its wealth, have transformed thought, relieved suffering, turned misery into comfort and saved innumerable lives, have been made by persons who were pursuing knowledge for its own sake, and who very seldom had the remotest guess as to the direction in which their work would lead them and their successors. They were what is called pure researchers, "studious porers over Nature's plan."

We hope that every public man will read these essays, including the distinguished politician who, *à propos* of the question as to whether fats and oils were to be regarded as contraband of war, announced that it had "recently been discovered that glycerine could be manufactured from fat." And we hope that every reader will draw, and remember, two inevitable inferences. The first inference is that in the modern world every public man, whether he know Greek or not, is hopelessly unfitted for any public post, in the Government itself or in any Government office, unless a very large part of his education has been education in science. The second inference is that the opportunities for scientific research should be made so great that no single person with an aptitude for research should be prevented from devoting his life to it, and the reward for great accomplishment in research should be so attractive that no person of aptitude, ability, and ambition should be tempted from it by the greater reward that can be gained in any other avocation. The Cambridge essayists state their case, but are very far from pressing it to its obvious conclusion. It is worth while, however, considering the following facts. At the present time there are in England five persons who have gained peerages and pensions of £5,000 a year because, after having earned large incomes at the Bar, they consented to enjoy salaries of £10,000 a year as Lord High Chancellor. The historical justification for these rewards is, we believe, that a peerage, a salary of £10,000 a year, and a pension of £5,000 a year were necessary to secure the impeccable honesty of lawyers. There is at the present time no single person in England upon whom a peerage has been conferred because of distinction in scientific discovery. There has never been in the history of England a pension of £5,000 a year given to a scientific researcher. And it is the lawyers who have muddled the history of England, and the scientific researchers who have added to the power and possibilities of mankind.

### REPORT OF THE RADIUM INSTITUTE.

**The Radium Institute, London.** A Report of the Work from Jan. 1, 1915, to Dec. 31, 1916. Published with the authority of the Committee, Riding House Street, Portland Place, W. 1.

THERAPEUTICS, like any other branch of knowledge and more than most other branches, shows in its history plenty of evidences of human fallibility. What contemporary fame, and what speedy oblivion, have attended divers regimens, treatments, "healings," "cures," courses! In many directions nowadays progress has consisted in but disburdening ourselves of error and in knowing our own ignorance; and this until lately held good of cancer. Those violet leaves, or that *eau Maiche* or "blue electricity," vouched for—strange association—by some Lady Bountiful or dissenting minister, have been just straws to be clutched at by the dying, eager to spend their money in return for a moment's hope. It seems strange to be able to believe, as reasonable physicians are now able, that a treatment has been found with real therapeutic action *in vivo* on the cancer cell, and unattended with damage to the patient's tissues when employed by an experienced hand. Not, of course, that many practical drawbacks have not cropped up to exercise the wits of specialists for many years to come. Epitheliomata of the buccal, laryngeal, and pharyngeal mucous membranes, says Mr. Hayward Pinch on the very first page of the current report of the Radium Institute in Portland Place, still prove very troublesome; the final result is usually disappointing. Also, "Radium should never be used as a substitute for operative interference in the treatment of early cases of mammary cancer, and should be resorted to only when the disease is inoperable." This good rule is only departed from, apparently, in the case of early rodent ulcer, which of all forms of malignant disease is the most amenable to the action of radium. It is also, one easily reflects, about the least serious form. That is one of life's little ironies. Possessing a good specific remedy, mercury, for syphilis, we discover salvarsan, which is only slightly superior. What we should have liked would have been a specific for tubercle. But then, as Empedocles taught, man errs because he dreams the world does but exist his welfare to bestow: the farmer, in short, will always grumble at the weather, and so some cancer patients will grumble in the same way at radium. The wise medical man, imitating—it is true with greater initial advantage—Nature's "equal mind," will console himself with the proof the radiologist gives him of sarcoma—yes, formidable sarcoma—when taken early shrinking away before radium emanation, causing a slight systemic upset while doing so, quite after the fashion quacks and sympathetic lay friends used to describe out of their imaginations as the result of rubbishy eighteenth-century "cures"; and leaving the patient really rid of his disease. For details, as the reviewers and analysers of technical literature say, see the original. This Institute is bound to hold a position of rare authority in the field of radium therapy, and rightly so. It was early recognised that so costly a means must be administered by communal effort, like the Navy or the Post Office, in place of here and there a medical man rather richer than his fellows hoarding a few hundred pounds' worth of the stuff. We should have liked to see evidence of more provision for research; but, then, our "equal mind" may mislead us, not having cancer, and make us forget the huge demand there must be for help for patients whose vitals are being gnawed at, and for whom time is everything. Seemingly, too, the Institute works day and night, Dr. Lynham taking charge during the latter period. And the second excuse is the one so valid, general, and topical that it needs no mention.

### Reviews in Brief.

**Minor Surgery and Bandaging.** By H. Morriston Davies, M.D., F.R.C.S. (London: J. and A. Churchill. Sixteenth Edition. 1917. Pp. 476. Price 8s. 6d. net.)

The new edition of this well-known text-book, originally written by Heath and afterwards edited by Bilton Pollard, is chiefly noticeable for the addition of a chapter on "War Surgery." Otherwise the changes from the last edition (1914) are not great; they scarcely could be in view of the pitch of excellence to which constant re-editing has brought this most valuable work. As for the chapter on war injuries, elementary principles are alone expounded, very wisely and judiciously, for space is of necessity limited. Experience shows, however, that the emphasis of elementary principles is by no means supererogatory; for there are surgeons who in their zeal for some new device or some revived hypothesis are led into forgetting that "war surgery" is but the application of fundamental surgical principles to certain injuries which in civil practice are rare, though not unknown. The only criticism of this chapter which can be justly advanced is that the author "sits on the fence" too much over the relative advantages of different systems of treating suppurating wounds; he mentions them all, but is not sufficiently dogmatic on their relative merits as determined by his own experience. Otherwise we have nothing but praise for a book which is one of the classics of British surgical literature.

**A Regimental Surgeon in War and Prison.** By Captain R. V. Dolbey, F.R.C.S., R.A.M.C. (London: John Murray. Price 5s. net.)

The author of this book of personal experiences during the first year of the war is a surgeon from British Columbia, who happened to be in England when the war broke out and took a temporary commission in the R.A.M.C. right away. He reached the Front just about the end of the retreat from Mons, apparently, and was taken prisoner near La Bassée some two months later, having seen much and varied fighting in the meanwhile. His account of the battles at the Aisne and round La Bassée is vivid and full of interest, as is also that of his experiences as a prisoner in Germany, where he was detained, in flat violation of the Geneva Convention, for about nine months. Though Captain Dolbey has a distinct gift for writing, we suspect that he is not a practised hand at it. Any experienced journalist could have re-arranged his far too lengthy paragraphs to much greater advantage; and the effect of some of his more ambitious flights is quite spoilt by the obvious effort and labour of them. Still, personal experiences (especially of the early part of the war and of the unspeakable horrors of the German treatment of our prisoners) are all valuable, and it is only plain truth to state that Captain Dolbey has a distinctly unusual personality, is obviously a most intelligent observer as well as a distinguished surgeon, and has produced a thoroughly straightforward and evidently honest account of very moving adventures gallantly encountered and sustained. It is difficult to understand why so expert a surgeon was ever allowed to become medical officer to an infantry battalion. His proper place was a base hospital or a casualty-clearing station; but that is a criticism of the Army Medical Service, not of the author of a very excellent book.

**Acute Appendicitis.** By C. Hamilton Whiteford, M.R.C.S., L.R.C.P. Late Specialist in Surgery, Military Hospital, Devonport. (London: Harrison and Sons, 45 Pall Mall, S.W. 1917. Pp. 72. Price 5s. net.)

The experiences of individual surgeons are always welcome, and into this little volume Mr. Hamilton Whiteford states that he has compressed the experiences of a quarter of a century. The conclusions at which he has arrived

**THE INSTITUTIONAL LIBRARY—(*continued*).**

are clearly stated, and will be useful to many medical practitioners who are obliged to undertake operations of emergency, although they do not aspire to the rank of surgeon. In the case of appendicitis, Mr. Whiteford lays especial stress upon the value of the pulse and respiration as a guide to the condition of the patient, and he is quite decided that immediate operation is the proper treatment, whilst the policy of drift or wait and see is fraught with danger. Practising, as he does, in the West Country, where the population is scattered, the distances are considerable, and the methods of locomotion are slow, he lays more stress upon the suppurative forms than is usual, and for this very reason the book is a valuable guide, because it teaches the country-side not to wait, but to make an early diagnosis so as to ensure prompt treatment. When this end has been attained it will not usually be necessary to make a double abdominal incision. The teaching is good, and the book can be heartily recommended.

**Cycling for Health, and Points for Cyclists.** By Sir FRANK BOWDEN, Bart., F.R.G.S. (Leicester and London : The Criterion Press, Ltd. Pp. 99. Price 1s.)

THIS is a pleasant and instructive little book written by one who, having retired in seemingly chronic ill-health after long service in a tropical country, took up cycling, under medical advice, as a cure for anæmia and general debility, and with returning physical vigour became first an enthusiastic cyclist and later a successful manufacturer of bicycles and tricycles. One whose working hours and hours of relaxation have been so much taken up with one subject may be excused when he collects a number of medical opinions favourable to his hobby and his business, but there is a certain danger in giving too wide publicity to the statement that cycling "has a wonderful effect in cases of gout, rheumatism, partial paralysis, stiffness in the joints of the legs in a man seventy years old, obesity, pulmonary consumption, and even heart disease." The author, it is true, advises that in the two latter cases patients should not ride without the advice of an experienced cycling physician, but he is on safer ground in his excellent chapters on how to choose a cycle, what to eat and drink when you are riding it, and how to keep your machine in order.

**The Expectant Mother.** By C. L. NORMAN. (London : The Scientific Press, Ltd., 28 and 29 Southampton Street, Strand, W.C. 2. Price 2d., by post 3d.)

THIS small and simply expressed pamphlet is intended for the instruction of the expectant mother of the labouring classes, whose means are limited and whose time and education do not permit of her deriving benefit from the study of larger and more expensive manuals on this subject. Though of modest dimensions, there is much sound teaching packed into Miss Norman's pages. Undoubtedly her experiences as a Queen's Nurse, a Certified Midwife, and a Health Visitor have well fitted her to undertake the *rôle* she here fulfils; and she has put her training and knowledge of conditions of life amongst the poorer classes to good account.

